# Unanswered questions when human karyotyping shows a supernumerary chromosome

**DOI:** 10.1038/s41437-025-00799-1

**Published:** 2025-09-30

**Authors:** Thomas Liehr, Sigrid Fuchs

**Affiliations:** 1 Jena University Hospital, Friedrich Schiller University, Institute of Human Genetics, Jena, Germany; 2 Institute of Human Genetics, University Medical Center Hamburg-Eppendorf, Hamburg, Germany

**Keywords:** Cytogenetics, Genomics

Nowadays, many different cytogenomic approaches can be used to obtain hints on chromosomal changes in primary material or cell lines. Chromosome microarrays (CMA) and optical genome mapping (OGM) can provide evidence of gains or losses in copy numbers affecting parts of or entire chromosomes. OGM and long-range sequencing can also deliver evidence of structural aberrations. However, the only approach suitable for unambiguous, single-cell-oriented characterization of numerical and structural rearrangements is karyotyping, which can be performed by banding cytogenetics and molecular cytogenetic techniques on metaphase chromosomes (Wang and Liehr 2025). It is regrettable that this needs to be mentioned, but in stem cell research, cytogenetic analyses of a cell line are already falsely treated as equivalent to CMA (Ceschin et al. 2023).

The latter shows a certain ignorance and disregard for “old-fashioned” cytogenetic chromosome analyses, which are considered fundamentally outdated by newcomers to the field as an approach that is over 60 years old (Akkari 2023). As the authors of this article examine hundreds of diagnostic cases (molecular) cytogenetically every year, we would like to emphasize the unique possibilities of these techniques and highlight some unanswered questions that arise when a supernumerary chromosome is found in cytogenetic diagnostics.

## Trisomies and trisomic rescue

The presence of a supernumerary chromosome is the most common constitutional anomaly in humans. In addition to viable trisomies of chromosomes 13, 18, 21, and X, up to 30% of all pregnancies are trisomic. Since only about 0.6% of newborns have a trisomy, about 98% of trisomic pregnancies are terminated naturally (Blue et al. 2019).

**Fig. 1:**
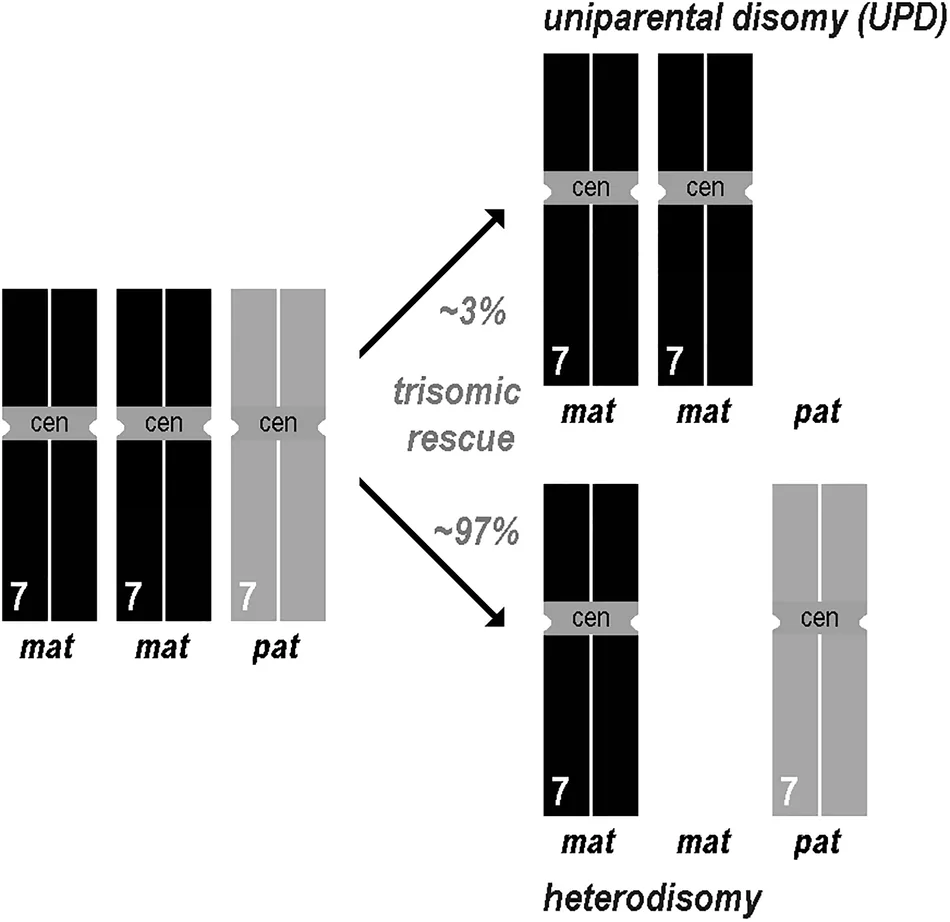
Schematic depiction of trisomic rescue; initiation, how often it is happening and how it works exactly are unknown. In case a cell identifies a trisomy (in this example of chromosome 7) and initiates trisomic rescue, then it identifies in ~97% of the cases the extra copy which comes (in this example) from the mother and removes this chromosome from the nucleus a normal fetus can develop now, as it will be heterodisomic for all chromosomes. In the remainder ~3% a uniparental disomy (UPD) is the result of the trisomic rescue; in this example a UPD(7)mat, which leads to Silver Russel syndrome.

However, it is known that a small proportion of trisomy cases can be traced back to a normal diploid constitution by trisomic rescue (Balbeur et al. 2016; Liehr 2024a). The latter term stands for a phenomenon known and regularly observed, that e.g., a zygote/an early embryo with trisomy 7 can find out about its aneuploidy condition and remove one of the three copies of chromosome 7 from its nucleus. Practically nothing is known yet how this ‘trisomic rescue event’ is initiated, regulated and how it proceeds. This means that the cells of the early embryo may have several hitherto unknown capabilities, which are listed in Table 1 together with the evidence on which these ideas are based; Fig. 1 shows corresponding examples.

**Table 1:** Unexplained capabilities of early embryonic cells concerning trisomic rescue.

Capabilities of early embryonic cells	Observations indicating specific capabilities	References
(1) The cell can recognize a trisomy of a specific chromosome—for example, the cell has one copy of chromosome 7 from the father and two copies from the mother (Fig. 1)	Trisomy rescue is not always complete; this means that successful, complete trisomy rescue results in a normal disomic cell. In rare cases, however, rescue is incomplete, meaning that a remnant of the copy of the extra chromosome to be eliminated remains. In this case, a small supernumerary marker chromosome (sSMC) is present. In our example, an sSMC from chromosome 7 would be present—normally, the sSMC would consist of pericentric material and lead to a small trisomy of the affected region	Balbeur et al. 2016; Liehr 2023 Liehr 2024a
(2) The cell can select one of the three chromosomes 7 and eliminate this copy from the genome (Fig. 1)		
(3) In most cases, they select one of the chromosomes that comes from the same parent for “trisomic rescue": in our example, chromosome 7, which comes from the mother (Fig. 1)	~97% of sSMCs have a biparental origin of their sister chromosomes and only ~3% are associated with uniparental disomy (UPD) – in our example, maternal UPD(7)	Liehr 2023 Liehr 2025a Liehr 2025b
(4) When such a “trisomic rescue” occurs, the embryo develops preferentially from a rescued cell, while the cells that still carry the trisomy preferentially form the placenta	At least 2% of prenatal cases detected by non-invasive prenatal testing (NIPT) are false positives, as NIPT tests screen for trisomies of the placenta	Liehr 2024b

The realization that each cell keeps the parental genome separate throughout life—most likely due to the never completely dissolved connection of the spindle apparatus from each haploid genome—could be a first step toward understanding how cells count their chromosomes and distinguish the parental origin of a trisomic chromosome with high accuracy (Weise et al. 2016).

**Fig. 2:**
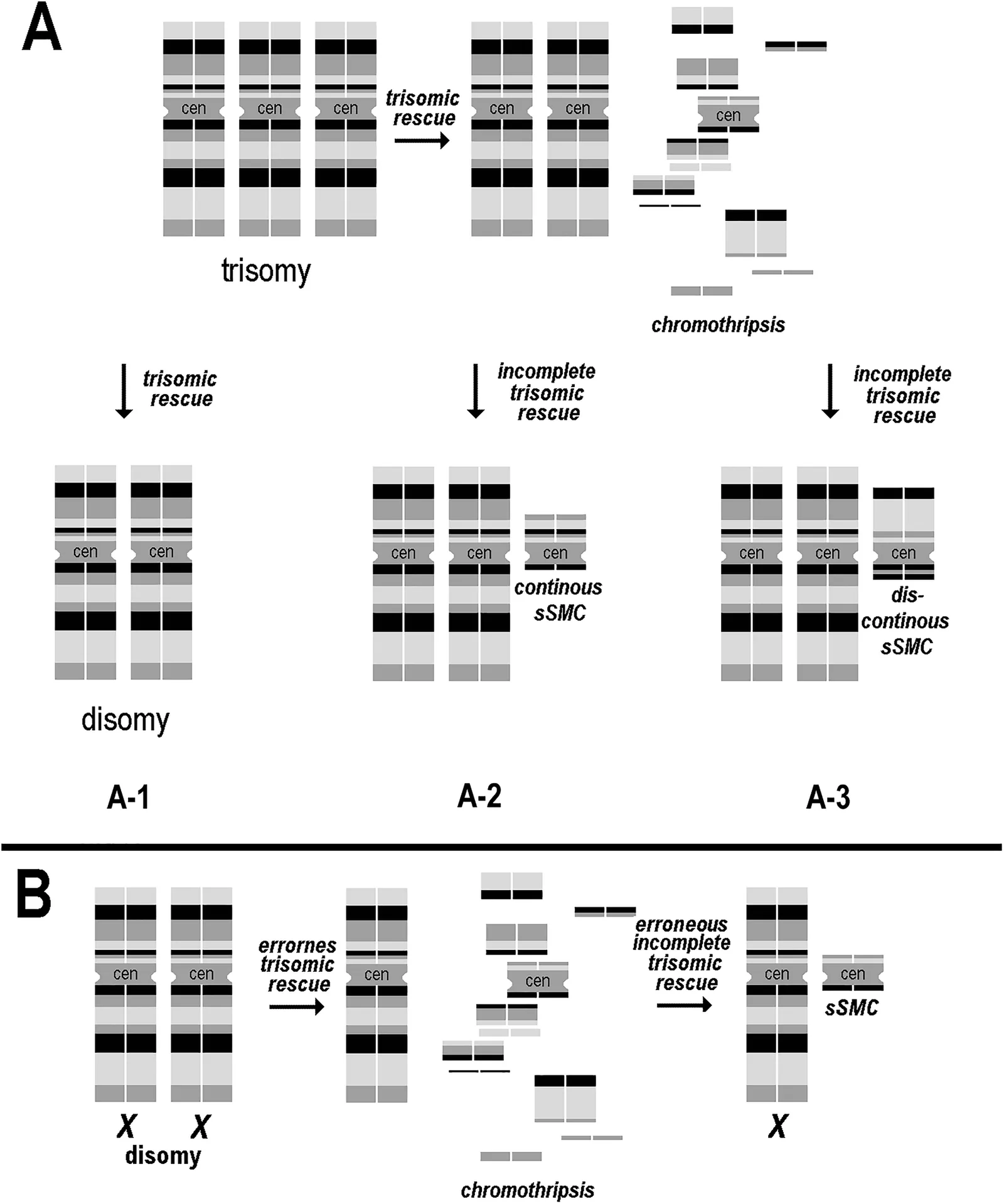
Different forms of trisomic rescue – all suggested to be based on chromothripis or similar events – are shown. **A-1** Successful trisomic rescue leading to a disomic situation. **A-2** Incomplete trisomic rescue leading to a continuous sSMC. **A-3** Incomplete trisomic rescue leading to a discontinuous sSMC. **B** Erroneous incomplete trisomic rescue of an X-chromosome leading to a karyotype 46,X,+mar(X).

There is also evidence that trisomic rescue (Fig. 2A-1) is partly caused by chromothripsis, chromoanasynthesis, and/or chromoplexia (Liehr 2023, 2024a). These three terms describe one step complex chromosomal rearrangements being associated with genomic instability. Chromothripsis is associated with localized fragmentation and subsequent balanced random reconstruction of a chromosome. Chromoanasynthesis leads to rearrangements and changes in copy number and is replication-based. Finally, chromoplexia results in “chains” of rearranged chromosomes due to a series of translocations and deletions.

## Formation of small supernumerary marker chromosomes (sSMCs)

Small supernumerary marker chromosome (sSMCs) are found additionally to an otherwise normal constitutional karyotype in ~0.044% of newborns. They lead to clinical problems in 30% of the cases and are without obvious consequence for the phenotype in the remainder 70%. sSMCs are found in prenatal and postnatal cases with and without clinical symptoms and in infertile patients. Their genotype-phenotype correlation is complicate, as mosaicism, imprinting effects (uniparental disomy) or genetic content of the sSMC influence their clinical effects (Liehr 2023).

**Fig. 3:**
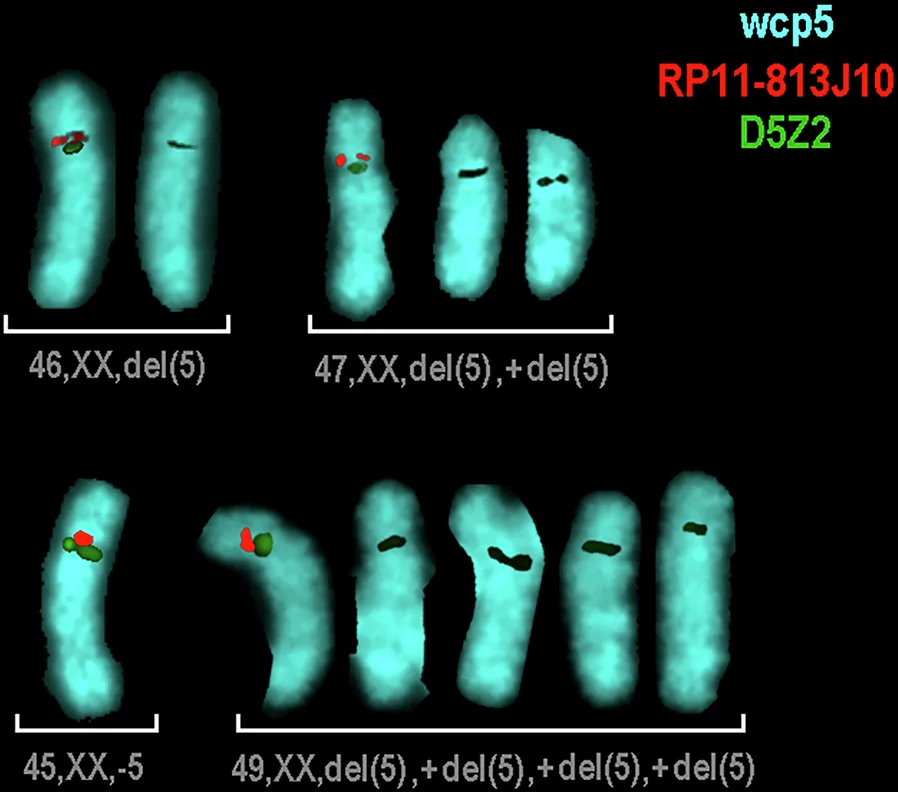
FISH result of a case with a mosaic karyotype mos 46,XX,del(5)[15]/45,XX,-5[7]/47,XX,del(5),+del(5)[2]/48,XX,del(5),+del(5),+del(5)[2]/49,XX,del(5),+del(5),+del(5),+del(5)[1].arr[GRCh37] 5p12p11(43,677,854-46,100,367)x1. Only (derivatives) of chromosome 5 are shown. The whole chromosome paint for chromosome 5 (wcp5) in blue helped in identifying chromosomes 5; the bacterial artificial chromosome probe RP11-813J10 (red) was absent in the derivative chromosomes 5; the region covered by the centromeric probe D5Z2 (green) was partially deleted on the derivative chromosome 5. Irrespective of total chromosome numbers, per cell only one copy of a normal chromosome 5 was present.

In most cases, sSMCs are considered to be the result of incomplete trisomy rescue (Liehr 2023). Due to the different shapes of sSMCs as centric minute, ring and inverted duplication shaped, different mechanisms are suspected for their formation. In the case of inverted duplication shaped sSMCs, however, a so-called U-type exchange in a chromosome is suspected to be the cause of their formation (Liehr 2023). However, since there are such sSMCs that originate from one chromosome and those that originate from two different chromosomes, their mode of formation is not yet fully understood (Liehr 2023). The involvement of chromothripsis, chromoanasynthesis, and/or chromoplexia is clear at least for centric minute-shaped sSMCs (Fig. 2A-2), as discontinuous sSMCs have been described and are thought to arise through these mechanisms (Fig. 2A-3). However, chromothripsis, chromoanasynthesis, and/or chromoplexia could still be the common mechanism for the formation of all types of sSMCs. A recently described neocentric sSMC, which originates from 5 different chromosomes, is the most extreme example of a discontinuous chromosome (Weber et al. 2022) and shows also features typically observed for B chromosomes (Houben et al. in press). It is under discussion, if a small subset of sSMCs already can be considered as B-chromosomes or not (Liehr 2023).

sSMCs can also occur in patients with Turner syndrome. Such sSMCs usually originate from an X or Y chromosome, and the karyotype is written as 46,X,+mar. The formation of this type of sSMC must be attributed to erroneous trisomic rescue (Liehr 2024a). This means that, for some reason, the early embryonic cell must have considered an X or Y chromosome to be surplur, even though the cell had the correct chromosome count of 46 (Fig. 2B). At least in Y-chromosome-derived sSMCs, a discontinuous shape has been repeatedly observed in such Turner syndrome patients, suggesting chromothripsis. Open questions related to the formation of sSMCs are summarized in Table 2 under points (1) and (2).

**Table 2:** Unexplained capabilities and features of sSMCs.

Open questions	Features of sSMCs	References
(1) How are sSMCs formed? Maybe - Chromothripsis - U-type exchange - other mechanisms	There are - discontinuous sSMCs. - inverted duplicated sSMCs - complex, neocentric and multiple sSMCs; McClintock-mechansism	Liehr 2023
(2) Are there B-chromosomes among sSMCs?	Candidates for human B-chromosomes are - heterochromatic +inv dup(15) - sSMC with unknown origin - neocentric acro-p-derived sSMCs	Liehr 2023
(3) Why do blood cells with tetrasomy 12p lose their sSMC(12)? (4) Why are sSMCs preferentially lost in fast dividing tissues?	Tetrasomy 12 cases lose their sSMC(12) in peripheral blood Neocentric sSMC also show a tendency to be lost in blood cells	Schubert et al. 1997 Liehr 2023
(5) Which forces drive trisomy or sSMC mosaicism in different tissues?	- see Fig. 3 - A complex mosaic pattern was seen in an sSMC(1) case	Fickelscher et al. 2007
(6) Why does shape of sSMC influence its mitotic stability?	Influence of shape of sSMC on mitotic stability has been shown	Hussein et al. 2014
(7) Why are some sSMC cases present in more than one shape/ size?	Different shapes of sSMCs in the same patient has been repeatedly observed	Liehr 2009 Liehr 2025a

## Mosaicism and (partial) trisomies

A trisomy of a chromosome and the presence of an sSMC can be associated with mosaicism.

### Mosaicism and trisomies

In complete chromosome trisomies, mosaicism with a normal cell line is very rare – mosaic trisomy 8 and 21 occur in 1 in 25,000 to 1 in 50,000 and 1:16,670 to 1:141,670 live births (Wiśniewska and Mazurek 2002; Jackson-Cook 2011). Mosaic karyotypes such as mos 47,XX, +21[15]/46,XX[15] are associated with a better prognosis and a less severe Down syndrome phenotype in most cases (Jackson-Cook 2011). Figure 3 shows the molecular cytogenetic result of a rare case of mosaic polysomy (and monosomy) of chromosome 5 in a 12-year-old female with severe developmental and behavioral disorders (including aggressive behavior, speech impairment, and inability to walk), a prominent nose, microcephaly, and muscular hypotonia. Karyotype was mos 46,XX[15]/45,XX,-5[7]/47,XX,+5[2]/48,XX,+5,+5[2]/49,XX,+5,+5,+5[1]. CMA revealed a 2.5 Mb deletion in 5p12 to 5p11 (chr5(GRCh37):43,677,854-46,100,367), including the genes *FGF10* and *HCN1* (Milunsky et al. 2006). LADD syndrome (lacrimo-auriculo-dento-digital) and the allelic ALSG syndrome (aplasia of the lacrimal gland and salivary gland) are caused by corresponding gene deletions and/or missense mutations in *FGF10* (Milunsky et al. 2006). *HCN1* missense mutations cause early-onset developmental and epileptic encephalopathy-24 (DEE24) (Nava et al. 2014; Marini et al. 2018). FISH confirmed that the deletion on the derivative chromosome 5 included RP11-813J10 in 5p12 (chr5(GRCh37):43,952,430-44,148,420) and a part of the centromeric region covered by D5Z2-alpha-satellite probe. Cells with two chromosomes 5 had one normal and one derivative chromosome 5; cells with one chromosome 5 lost the derivative one; and all cells with tri-, tetra- or heptasomy 5 all had only derivative chromosomes 5 except for one normal copy. In this case, it can be speculated that the partial loss of the centromeric alpha satellite DNA D5Z2 led to an unstable centromere of the derivative chromosome. Thus, mitotic non-disjunction led to the postzygotic development of a viable mosaic of poly- and monosomic cells. Overall, the patient’s clinical symptoms can be attributed in part to the deletion detected by CMA and in part, especially due to the severity of the symptoms, to secondary aneuploidy. This case shows once again that CMA should always be supplemented by further cytogenetic investigations.

### Mosaicism and partial trisomies

In sSMC cases, mosaicism is observed in up to 50% of cases. However, since mosaics can vary between different tissues, there is not always a clear correlation between karyotype and phenotype (Liehr and Al-Rikabi 2019). In tetrasomy 12p (Pallister-Killian syndrome), sSMC(12) is barely detectable in peripheral blood cells, while it is present in (almost) all skin fibroblasts and other body cells. One of the most extreme examples is the case of a woman who was examined for infertility and otherwise had a normal phenotype, but was diagnosed with tetrasomy 9p in all peripheral blood cells. As a severe mental and developmental impairment would have to be expected with such a chromosome-set, in this case a much lower percentage of corresponding cells with tetrasomy 9p must be assumed in the remaining ~400 tissues of the probands body (Liehr and Al-Rikabi 2019).

Mosaicism can vary not only between tissues but also over the course of a lifetime (Liehr 2023). While inverted duplication shaped sSMCs are mitotically relatively stable, centric minute and ring shaped ones tend to be lost (Hussein et al. 2014). Others may exhibit more than one shape per patient (Liehr 2009). It should also be noted that UPD is primarily due to a rescued chromosomal imbalance. Therefore, it is not surprising that, according to Liehr (2025b), 554/6260 (~9%) of reported UPD cases exhibit mosaic trisomy. 89 of the 554 cases (16%) with initial mosaic trisomy carry an sSMC. The open, unanswered questions deriving from the facts mentioned in this section are listed in Table 2 from points 3 onwards.

## Diagnostic dilemma

We might currently be experiencing a golden age of chromosomic research and diagnostics, as no other generation has had access to such cytogenomic tools as we do today (Wang and Liehr 2025).

Nevertheless, in modern human genetics one of its main goals is finding the one comprehensive approach, which replaces all others, including (molecular) cytogenetics (Akkari 2023). That this may not be a wise decision is demonstrated by the following two sSMC cases, in which banding cytogenetics, molecular cytogenetics, and CMA were necessary to make a comprehensive and unambiguous diagnosis and avoid misleading results. The first case (with infertility) was initially investigated using banding cytogenetics, which revealed the presence of an sSMC. Afterwards, CMA was performed, which provided evidence of a centromere-near euchromatic duplication of 1.54 Mb in 6q11.1. FISH showed that the sSMC actually originated from a completely different chromosome (it was an inv dup(15)(q11.1)), and the CMA result just had identified a benign CNV that was due to an intrachromosomal duplication on one of the two chromosomes 6. Even stranger was the second case: A parent of a relatively mildly affected carrier of an sSMC contacted the Jena laboratory and asked for advice. The de novo sSMC was present in the affected child in its peripheral blood in a mosaic pattern (~20%). Nevertheless, a CMA was performed next, which, as expected, did not yield any meaningful results due to the low mosaicism. Instead of further clarifying the origin of the sSMC, which was incorrectly assumed to be heterochromatic because the CMA had not identified a CNV, a whole exome analysis was performed. In this analysis, a previously unknown variant was found in a gene that, according to protein structure prediction programs, suggested a possible phenotypic effect. However, the latter did not really match the child’s symptoms. After FISH testing, the sSMC could be characterized as a derivative of chromosome 19, which included the known triplo-sensitive regions and explained the patient’s symptoms.

## Conclusion

Overall, this perspective paper shows that many questions remain unanswered in human genetic and chromosomic research, particularly in cases involving trisomy and sSMC. Basic and clinical research on the issues mentioned above should be prioritized, as from the answers obtains both will benefit: “pure science” and application of knowledge in counseling and clinical practice. In addition, human genetics must react quickly and reevaluate old approaches falsely classified as outdated. Otherwise, (molecular) cytogenetic expertise will be lost forever over the next decade.

## References

[CR1] Akkari Y (2023) Science that lasts a lifetime. The Pathologist. https://thepathologist.com/issues/2023/articles/jun/science-that-lasts-a-lifetime. Accessed 05 June 2025.

[CR2] Balbeur S, Grisart B, Parmentier B, Sartenaer D, Leonard PE, Ullmann U et al. (2016) Trisomy rescue mechanism: the case of concomitant mosaic trisomy 14 and maternal uniparental disomy 14 in a 15-year-old girl. Clin Case Rep. 4:265–271. 27014449 10.1002/ccr3.501 PMC4771849

[CR3] Blue NR, Page JM, Silver RM (2019) Genetic abnormalities and pregnancy loss. Semin Perinatol 43:66–73. 30638594 10.1053/j.semperi.2018.12.002 PMC8272960

[CR4] Ceschin II, Ceschin AP, Joya MS, Mitsugi TG, Nishikawa LK, Krepischi AC et al. (2023) Functional assessment of donated human embryos for the generation of pluripotent embryonic stem cell lines. Reprod Biomed Online 46:491–501. 36737274 10.1016/j.rbmo.2022.11.020

[CR5] Fickelscher I, Starke H, Schulze E, Ernst G, Kosyakova N, Mkrtchyan H et al. (2007) A further case with a small supernumerary marker chromosome (sSMC) derived from chromosome 1-evidence for high variability in mosaicism in different tissues of sSMC carriers. Prenat Diagn 27:783–785. 17546703 10.1002/pd.1776

[CR6] Houben A, Fuchs J, Banaei-Moghaddam AM, Chen J, Kim G, Liu T (in press) Does chromoanagenesis play a role in the origin of B chromosomes? Heredity (Edinb) 10.1038/s41437-025-00758-w PMC13545249 40253498

[CR7] Hussein SS, Kreskowski K, Ziegler M, Klein E, Hamid AB, Kosyakova N et al. (2014) Mitotic stability of small supernumerary marker chromosomes depends on their shape and telomeres - a long term in vitro study. Gene 552:246–248. 25245454 10.1016/j.gene.2014.09.041

[CR8] Jackson-Cook C (2011) Constitutional and acquired autosomal aneuploidy. Clin Lab Med 2011 31:481–511. 10.1016/j.cll.2011.08.002 PMC3269043 22118733

[CR9] Liehr T (2009) Small supernumerary marker chromosomes (sSMCs): a spotlight on some nomenclature problems. J Histochem Cytochem 57:991–993. 19654102 10.1369/jhc.2009.954370 PMC2762887

[CR10] Liehr T (2023) Small supernumerary marker chromosomes. Basics. Epubli, Berlin

[CR11] Liehr T (2024a) All you need to know about uniparental disomy. UPD and imprinting. Epubli, Berlin.

[CR12] Liehr T (2024b) Noninvasive prenatal testing (NIPT) results are less accurate the later applied during pregnancy. Taiwan J Obstet Gynecol 63:892–895. 39481998 10.1016/j.tjog.2024.05.027

[CR13] Liehr T (2025a) Small supernumerary marker chromosomes. https://cs-tl.de/DB/CA/sSMC/0-Start.html. Accessed 05 June 2025.

[CR14] Liehr T (2025b) Cases with uniparental disomy. https://cs-tl.de/DB/CA/UPD/0-Start.html. Accessed 05 June 2025.

[CR15] Liehr T, Al-Rikabi A (2019) Mosaicism: Reason for normal phenotypes in carriers of small supernumerary marker chromosomes with known adverse outcome. A systematic review. Front Genet 10:1131. 31781176 10.3389/fgene.2019.01131 PMC6859531

[CR16] Marini C, Porro A, Rastetter A, Dalle C, Rivolta I, Bauer D, Oegema R, Nava C, Parrini E, Mei D, Mercer C, Dhamija R et al. (2018) HCN1 mutation spectrum: from neonatal epileptic encephalopathy to benign generalized epilepsy and beyond. Brain 141:3160–3178. 30351409 10.1093/brain/awy263

[CR23] Milunsky JM, Zhao G, Maher TA, Colby R, Everman DB (2006) LADD syndrome is caused by FGF10 mutations Clin Genet 69:349–354 16630169 10.1111/j.1399-0004.2006.00597.x

[CR17] Nava C, Dalle C, Rastetter A, Striano P, de Kovel CGF, Nabbout R, Cances C, Ville D, Brilstra EH, Gobbi G, Raffo E, Bouteiller D et al. (2014) De novo mutations in HCN1 cause early infantile epileptic encephalopathy. Nature Genet 46:640–645. 24747641 10.1038/ng.2952

[CR18] Schubert R, Viersbach R, Eggermann T, Hansmann M, Schwanitz G (1997) Report of two new cases of Pallister-Killian syndrome confirmed by FISH: tissue-specific mosaicism and loss of i(12p) by in vitro selection. Am J Med Genet 72:106–110. 9295085 10.1002/(sici)1096-8628(19971003)72:1<106::aid-ajmg21>3.0.co;2-u

[CR19] Wang Y, Liehr T (2025) The need for a concert of cytogenomic methods in chromosomic research and diagnostics. Genes (Basel) 16:533. 40428355 10.3390/genes16050533 PMC12111664

[CR20] Weber A, Liehr T, Al-Rikabi A, Bilgen S, Heinrich U, Schiller J et al. (2022) The first neocentric, discontinuous, and complex small supernumerary marker chromosome composed of 7 euchromatic blocks derived from 5 different chromosomes. Biomedicines 10:1102. 35625839 10.3390/biomedicines10051102 PMC9138958

[CR21] Weise A, Bhatt S, Piaszinski K, Kosyakova N, Fan X, Altendorf-Hofmann A et al. (2016) Chromosomes in a genome-wise order: evidence for metaphase architecture. Mol Cytogenet 9:36. 27123045 10.1186/s13039-016-0243-y PMC4847357

[CR22] Wiśniewska M, Mazurek M (2002) Trisomy 8 mosaicism syndrome. J Appl Genet 43:115–118. 12084977

